# Case Report: A Novel Compound Heterozygous Mutation of the *FRMD4A* Gene Identified in a Chinese Family With Global Developmental Delay, Intellectual Disability, and Ataxia

**DOI:** 10.3389/fped.2021.775488

**Published:** 2021-11-17

**Authors:** Yuhua Pan, Xiaoling Guo, Xiaoqiang Zhou, Yue Liu, Jingli Lian, Tingting Yang, Xiang Huang, Fei He, Jian Zhang, Buling Wu, Fu Xiong, Xingkun Yang

**Affiliations:** ^1^School of Stomatology, Nanfang Hospital, Southern Medical University, Guangzhou, China; ^2^Affiliated Foshan Maternity & Child Healthcare Hospital, Southern Medical University, Guangzhou, China; ^3^Department of Medical Genetics, Experimental Education, Administration Center, School of Basic Medical Sciences, Southern Medical University, Guangzhou, China; ^4^Guangdong Provincial Key Laboratory of Single Cell Technology and Application, Guangzhou, China; ^5^Department of Fetal Medicine and Prenatal Diagnosis, Zhujiang Hospital, Southern Medical University, Guangzhou, China

**Keywords:** global developmental delay (GDD), compound heterozygous missense mutations, corpus callosum anomaly, *FRMD4A*, intellectual disability

## Abstract

**Background:** FERM domain-containing protein 4A (*FRMD4A*) is a scaffolding protein previously proposed to be critical in the regulation of cell polarity in neurons and implicated in human intellectual development.

**Case Presentation:** We report a case of a 3-year-old boy with corpus callosum anomaly, relative macrocephaly, ataxia, and unexplained global developmental delay. Here, compound heterozygous missense mutations in the *FRMD4A* gene [c.1830G>A, p.(Met610Ile) and c.2973G>C, p.(Gln991His)] were identified in the proband, and subsequent familial segregation showed that each parent had transmitted a mutation.

**Conclusions:** Our results have confirmed the associations of mutations in the *FRMD4A* gene with intellectual development and indicated that for patients with unexplained global developmental delay, the *FRMD4A* gene should be included in the analysis of whole exome sequencing data, which can contribute to the identification of more patients affected by this severe phenotypic spectrum.

## Introduction

Global developmental delay (GDD) and intellectual disability (ID), which constitutes 3% of the pediatric population (1, 2), influences the physical and mental health of patients and their lives, bringing about heavy burdens for families and societies. Because the etiological diagnoses of GDD and ID overlap, the investigations leading up to a definitive diagnosis for these two diseases are naturally similar. Early detection and diagnosis are very important to the initiation of rehabilitative treatment as soon as possible. Genetic defects are responsible for the development of intellectual impairment in ~25% of patients, resulting in structural and/or functional defects of the central nervous system (3).

A previous study has described a family with a homozygous mutation in the *FRMD4A* gene, which resulted in a syndrome of congenital microcephaly, intellectual disability, and dysmorphism (4). In addition, *FRMD4A* polymorphisms have been suggested to be a genetic risk factor for Alzheimer's disease (5). We now report a novel compound heterozygous mutation in the *FRMD4A* gene in a child who has a GDD, ID, and corpus callosum anomaly.

## Case Presentation

### Clinical Examination

**Figure 1:**
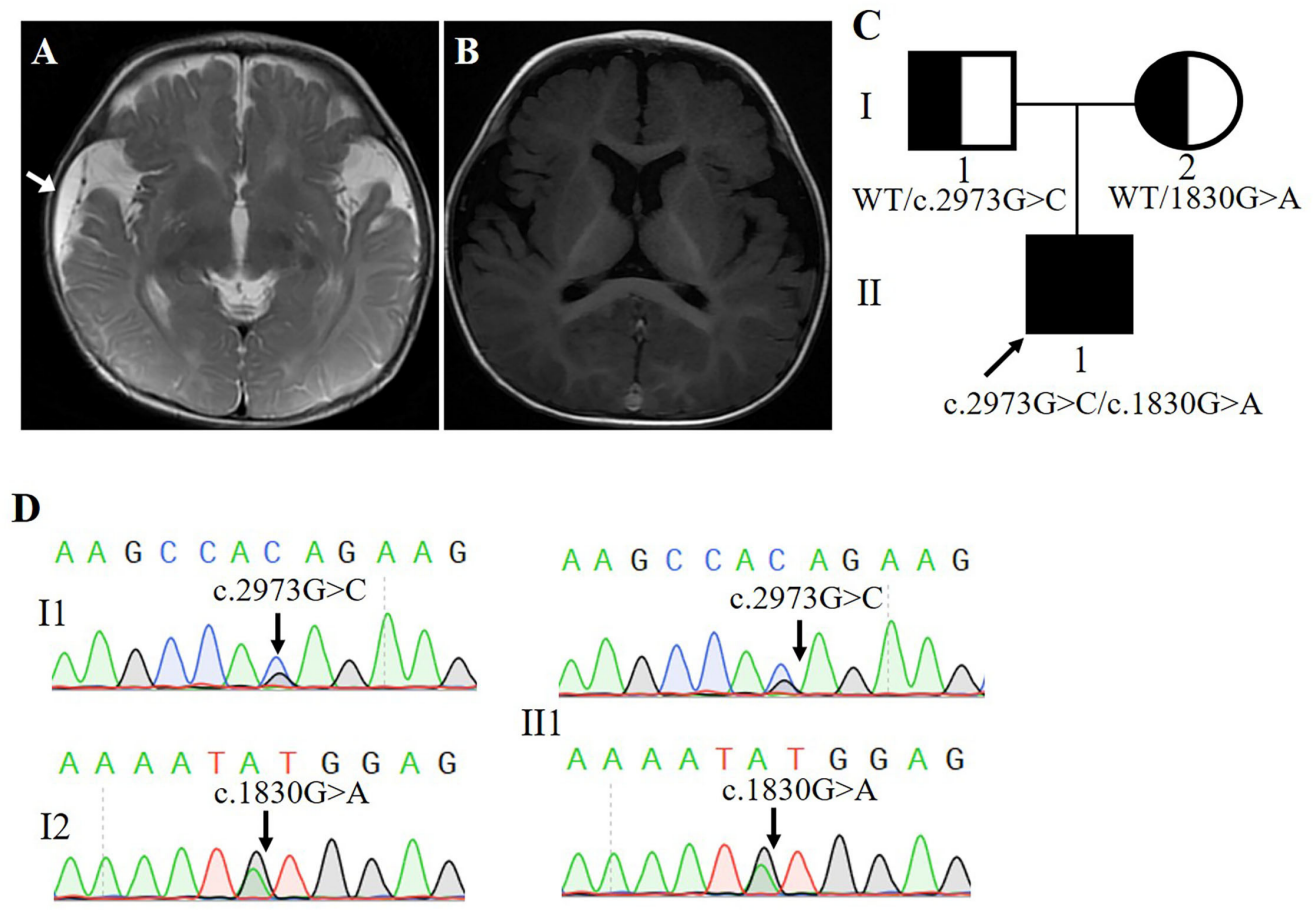
**(A,B)** Magnetic resonance imaging of the patient's head (Arrow points to area of subdural effusion). **(C)** Poband (II-1) is shown. The squares represent the proband, and his father. The circle represents the mother. **(D)** Sanger sequencing showing two alleles of the proband and his parents, respectively.

Our subject was a 3-year-old boy born at 36 weeks' gestation and the first child of healthy non-consanguineous Chinese parents. His birth weight was 3,600 g. At 6 months of age, his head circumference was 48.3 cm (+0.3 SD). At 11 months of age, his head circumference was 51 cm (+0.35 SD). The clinical examination results showed that the patient often had straightening of bilateral lower limbs, lasting for several seconds, and the frequency was uncertain, which was suggestive of non-epileptic seizure, with low muscular tension in his limbs. Skull magnetic resonance imaging (MRI) of diffusion-weighted images (DWI) of the genu of corpus callosum and the splenium of corpus callosum presented abnormal high-signal shadow, slightly decreased cerebral white matter, and right frontal-temporal-parietal subdural effusion (Figure 1A). MRI re-examination disclosed a widened extracerebral space of bilateral temporal and parietal lobes (Figure 1B). The history and clinical examination findings were suggestive of either a genetic or a brain development abnormality.

### Molecular Investigation

Blood samples were collected and sent for genetic testing and molecular studies after obtaining informed written consent from the parents. The pedigree was drawn after obtaining detailed family information (Figure 1C).

Whole-exome sequencing results showed that two previously unreported missense mutations in the *FRMD4A* gene (NM_018027), namely, c.1830G>A, p.(Met610Ile) in exon 20, and c. 2973G>C, p.(Gln991His) in exon 23 were found to be the cause of brain development abnormality in the proband of the family (Table 1). Sanger sequencing showed that the patient inherited the p.(Gln991His) mutation from his father and the p.(Met610Ile) mutation from his mother, consistent with an autosomal recessive mode of inheritance (Figure 1D). These mutations were further confirmed by Sanger sequencing analysis. The primers used for this test were as follows: forward 1, 5′-CTTGTCTTGGCAACTGGGGA-3′ and reverse 1, 5′-GCGCTGCCTGAGATTTCCTA-3′; and forward 2, 5′-GCCACACTGAAAATGCCCTG and reverse 2, 5′-ACAGCAGATCATGGGGCTTC-3′.

**Table 1:** Molecular information of compound heterozygous mutation on chromosome 10 of *FRMD4A* family.

**Position**	**cDNA change**	**Amino acid change**	**PolyPhen2**	**SIFT**	**Mutation taster**	**Provean**	**ACMG**
Chr10:13702384 (GRch37.p13)	c.1830G>A	Met610Ile	Benign (0.001)	Tolerated (0.063)	Disease-causing	Neutral (−1.01)	Uncertain significance
Chr10:13696493 (GRch37.p13)	c.2973G>C	Gln991His	Damaging (0.992)	Damaging (0.000)	Disease-causing	Neutral (−0.2)	Uncertain significance

The p.(Met610Ile) mutation of the *FRMD4A* gene was predicted to be probably benign according to PolyPhen-2, SIFT, and Provean (Table 1), and this mutation was novel as it had not been previously reported and it was not present in dbSNP, HGMD, or Exome Variant Server. The p.(Gln991His) mutation of the *FRMD4A* gene was predicted to affect the features of the protein and to be disease-causing according to PolyPhen-2, SIFT, and Mutation Taster (Table 1). We also compared the clinical characteristics caused by mutations at different sites of he FRMD4A (Table 2).

**Table 2:** Summary of the clinical features of the present study and published case associated with *FRMD4A* mutation.

**References**	**Gender**	**Age**	**Population**	**Family history**	**Mutations in *FRMD4A***	**Exons**	**Ataxia**	**MRI**	**Other information**
Present study	Male	3 years	Han Chinese	—	c.1830G>A c.2973G>C	20 23	F0C6	Abnormal genu of corpus callosum and the splenium of corpus callosum	Macrocephaly
Fine D et al.	Male	6 years	Israeli Bedouin	F0C6	Homozygousc. 2134_2146dup13	22	F0C6	Partial to near full agenesis of corpus callosum and various degrees of hypoplasia of the vermis and cerebellum	Microcephaly

**Figure 2:**
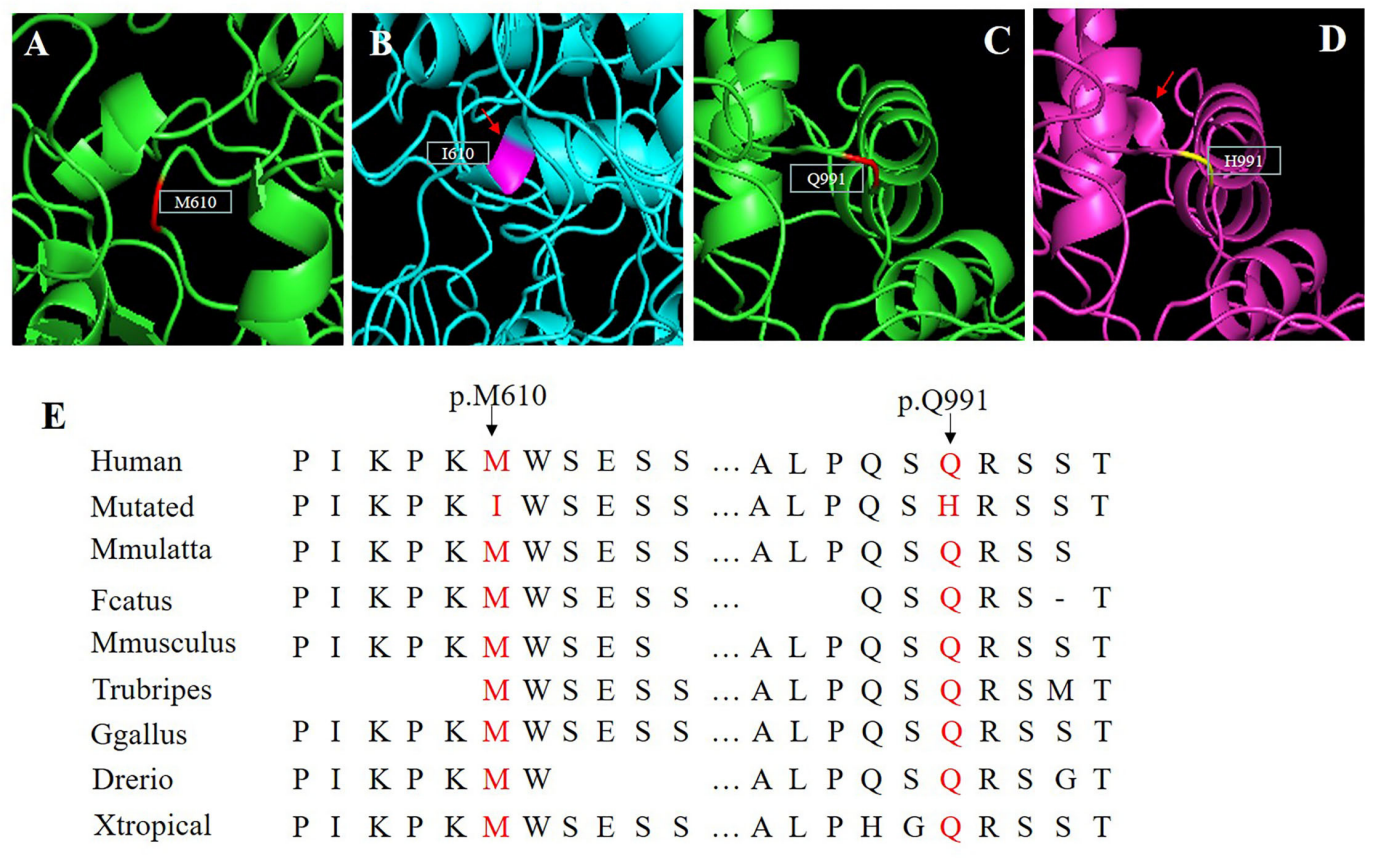
**(A–D)** Protein molecular models of wild type (upper) and *FRMD4A* mutations (lower) by I-TASSER. **(E)** Conservation analysis of the abnormal variation. Results indicate that both Met610 and Gln991 in the *FRMD4A* protein are highly conserved between different species.

A 3D model was constructed for the structural analysis of WT/MUT *FRMD4A* proteins to determine the pathogenicity of mutant *FRMD4A* according to I-TASSER. When amino acid 610 was changed to isoleucine, the side chains of *FRMD4A* also changed, and they tended to be longer than those of a structure in which amino acid 610 was methionine (Figures 2A,B). The mutated H911 protein had a different side chain than the wild-type protein as a result of an amide glutamine being substituted for a basic amino acid histidine (Figures 2C,D). Both p.(Met610Ile) and p.(Gln991His) mutations of the *FRMD4A* gene consisted of highly conserved amino acid residues among different species (Figure 2E), indicating their structural and functional importance. Therefore, the compound heterozygous mutations were predicted to affect the amino acid side chain. This might have disrupted *FRMD4A* function as well as interactions with other molecules and residues.

## Discussion and Conclusion

We described a young boy with a syndrome of global developmental delay, intellectual disability, relative macrocephaly, and non-epileptic seizure. In fact, many studies on global developmental delay and intellectual disability are usually accompanied by macrocephaly (6–8). However, no other mutations assumed to be consistent with the autosomal recessive inheritance pattern of the family were identified by whole-exome sequencing, except a new compound heterozygous mutation of the *FRMD4A* gene. Remarkably, by contrast, previously described patients with the *FRMD4A* mutation presented microcephaly (4), not macrocephaly. This different clinical feature represents a possible novel phenotypic trait involving the *FRMD4A* mutation. Although the phenotype characteristic of head circumference was inconstant, we conclude that the association of epilepsy, intellectual disability/global developmental delay and ataxia is a recurrent clinical pattern in cases with *FRMD4A* mutations. To our knowledge, our patient is only the second case of the syndrome caused by the *FRMD4A* mutation in the literature (4), and the first case reported compound heterozygosity.

The *FRMD4A* protein, a member of the FERM superfamily, is localized in the cytoplasm and the cytoskeleton, where it binds molecules in the undercoat of the cell-to-cell adherens junction (9). The protein encoded by the *FRMD4A* gene is involved in cell structure, transport, and signal transduction, and it plays a role in the regulation of cell polarity in epithelial cells and neurons (10, 11). In addition, *FRMD4A* is expressed in many tissues, with higher levels in the brain (12, 13). More importantly, the relationship between the *FRMD4A* protein and the brain is not only reflected in the syndrome of microcephaly and intellectual disability caused by its mutation (4), but also in the phenotypes that are observed in carriers of its genomic copy number mutations that increase the risk of schizophrenia (14). Interestingly, other studies have reported that *FRMD4A* mutations are regarded as a genetic risk factor for late-onset Alzheimer's disease in that they can disrupt tau secretion by activating cytohesin–Arf6 signaling (5, 11).

In epithelial cells, *FRMD4A* was demonstrated to act as a scaffolding protein, regulating cell polarity by connecting the Par3–Par6–aPKCζ complex to Arf6 signaling through cytohesin-1 (10). Par polarity complex signaling plays an important role in neuronal polarization (15) but also in membrane trafficking, including vesicular secretion (16). The fact that *FRMD4A* binds to both Par3 and the ARF6 guanine nucleotide exchange factor (cytohesin-1) suggests that *FRMD4A* mediates their interaction and that the Par3–*FRMD4A*–cytohesin-1 complex ensures accurate activation of the ARF6 protein (17). On the other hand, in HEK293t cells, *FRMD4A*, a genetic risk factor for late-onset Alzheimer's disease, modulates cellular release of tau through cytohesins and Arf6 (11).

In summary, a compound heterozygote in the *FRMD4A* gene was identified in a 3-year-old male patient with a severe neurological phenotype of unique dysmorphism, and a novel missense mutation was suspected. The results were confirmed by Sanger sequencing. The source of mutations was investigated by two-generation pedigree analysis. The current results broaden the spectrum of *FRMD4A* mutations responsible for the neurological phenotype, and they have important implications for molecular diagnosis, treatment, and genetic counseling in the future.

## Data Availability Statement

The original contributions presented in the study are included in the article/supplementary materials, further inquiries can be directed to the corresponding author/s.

## Ethics Statement

The studies involving human participants were reviewed and approved by the study was approved by the Ethics Committee of the Foshan Maternity & Child Healthcare Hospital to Southern Medical University. Written informed consent to participate in this study was provided by the participants' legal guardian/next of kin. Written informed consent was obtained from the individual(s) for the publication of any potentially identifiable images or data included in this article.

## Author Contributions

XY, XG, XZ, YL, JL, TY, and XH contributed to the acquisition and analysis of the clinical data. YP, FH, JZ, BW, and FX designed and performed the molecular analysis of the patient and patient's parents. All the authors contributed with the draft of the manuscript, and read and approved the final manuscript.

## Funding

This work was supported by the National Natural Science Foundation of China (82171713, 31970558 and 81870755), Foshan Science and Technology Bureau (2020001003953), Natural Science Foundation of Guangdong Province (2020A1515010308), and Project of Foshan Genetic Disease Precision Diagnosis Engineering Technology Research Center (2020001003953).

## Conflict of Interest

The authors declare that the research was conducted in the absence of any commercial or financial relationships that could be construed as a potential conflict of interest.

## Publisher's Note

All claims expressed in this article are solely those of the authors and do not necessarily represent those of their affiliated organizations, or those of the publisher, the editors and the reviewers. Any product that may be evaluated in this article, or claim that may be made by its manufacturer, is not guaranteed or endorsed by the publisher.
